# Estimation of the visual contribution to standing balance using virtual reality

**DOI:** 10.1038/s41598-023-29713-7

**Published:** 2023-02-14

**Authors:** Lorenz Assländer, Matthias Albrecht, Moritz Diehl, Kyle J. Missen, Mark G. Carpenter, Stephan Streuber

**Affiliations:** 1 Human Performance Research Centre, University of Konstanz, 78464 Konstanz, Germany; 2 Department of Computer and Information Science, University of Konstanz, 78464 Konstanz, Germany; 3 Department of Mathematics, University of Freiburg, 79110 Freiburg, Germany; 4 School of Kinesiology, University of British Columbia, Vancouver, V6T 2A1 Canada; 5 Department of Electrical Engineering and Computer Science, Coburg University of Applied Sciences and Arts, 96450 Coburg, Germany; 6 Zukunftskolleg, University of Konstanz, 78464 Konstanz, Germany

**Keywords:** Motor control, Sensorimotor processing, Sensory processing, Visual system

## Abstract

Sensory perturbations are a valuable tool to assess sensory integration mechanisms underlying balance. Implemented as systems-identification approaches, they can be used to quantitatively assess balance deficits and separate underlying causes. However, the experiments require controlled perturbations and sophisticated modeling and optimization techniques. Here we propose and validate a virtual reality implementation of moving visual scene experiments together with model-based interpretations of the results. The approach simplifies the experimental implementation and offers a platform to implement standardized analysis routines. Sway of 14 healthy young subjects wearing a virtual reality head-mounted display was measured. Subjects viewed a virtual room or a screen inside the room, which were both moved during a series of sinusoidal or pseudo-random room or screen tilt sequences recorded on two days. In a between-subject comparison of 10 $\times$ 6 min long pseudo-random sequences, each applied at 5 amplitudes, our results showed no difference to a real-world moving screen experiment from the literature. We used the independent-channel model to interpret our data, which provides a direct estimate of the visual contribution to balance, together with parameters characterizing the dynamics of the feedback system. Reliability estimates of single subject parameters from six repetitions of a 6 $\times$ 20-s pseudo-random sequence showed poor test–retest agreement. Estimated parameters show excellent reliability when averaging across three repetitions within each day and comparing across days (Intra-class correlation; ICC 0.7–0.9 for visual weight, time delay and feedback gain). Sway responses strongly depended on the visual scene, where the high-contrast, abstract screen evoked larger sway as compared to the photo-realistic room. In conclusion, our proposed virtual reality approach allows researchers to reliably assess balance control dynamics including the visual contribution to balance with minimal implementation effort.

## Introduction

Humans rely on sensory information to maintain balance^1^. Controlled perturbations of the sensory inputs are a powerful tool to investigate sensory integration and its deficits^2–6^. However, controlled sensory perturbations are difficult to implement, both in terms of hardware and analysis techniques. In our study, we propose and validate sensory perturbation experiments using a simple virtual reality (VR) setup. We further use model-based interpretations of the recorded data on a single subject level as proposed by Peterka^5^. We will show that a combination of VR experiments and model-based interpretations can reliably and easily assess the dynamics of the balance control mechanism including quantitative estimates of the visual sensory contribution (weighting factor).

One important focus of balance experiments is the assessment of sensory integration and its deficits to diagnose balance disorders^7,8^. Severe disorders become already visible during simple tasks such as standing with feet together and closing the eyes (Romberg stance)^9^. Instrumented balance tests, such as the assessment of spontaneous sway using force plate measurements, provide more detailed and objective information^8^. However, spontaneous sway measures do not contain sufficient information to separate internal control dynamics from the unknown noise properties inherent in sensory and motor systems^6^. It further does not provide insight into how the system deals with sensory conflicts, which occur in every day life when standing on soft surfaces or when viewing a moving visual scene (e.g. a train or bus). Thus, while providing valuable information about the state of the system spontaneous sway provides only limited information on sensory integration^6^. Changing sensory availability (firm and soft support, eyes open/closed, etc.) can be used to assess sensory integration to some extent^2^. An instrumented implementation based on sensory removal mostly used in clinical settings is the sensory organization test (SOT)^10,11^. Within three of the SOT conditions, the support surface and/or the visual scene are moved with a subject’s sway (’sway referencing’^12^). In these conditions, the sway referenced system does not accurately encode body position in space. Abnormal sway behavior or the inability to stand in such conditions indicates balance deficits and provides hints on the affected systems. The SOT score includes spontaneous sway and ’falls’ of subjects. While the SOT score is able to reveal balance deficits, it does not take into account interactions between multiple deficits and the complexity of sensorimotor interactions. Thus, it still provides insights into the state of the system, rather then revealing the systems dynamics and properties itself.

Systematically inducing sensory perturbations is a very powerful paradigm to investigate sensory integration and characterize the balance control mechanism itself^2–6^. For example, visual scene or support surface tilts can be used to induce subtle conflicts between sensory inputs. The central nervous system is not able to resolve the sensory conflicts perfectly. The imperfections lead to erroneous interpretations of visual scene movements as self-motion. Erroneously sensed self-motion leads to ’corrective’ muscle contractions, thereby inducing body sway. The relation of conflict and evoked sway is a rich source of information which can be used to characterize and quantitatively model the underlying sensory integration mechanism^1,6^.

In the last three decades, a comprehensive framework for the interpretation of tilt perturbation data has been developed^1,5,6,13–15^. The approach uses systems-identification tools from control theory that reduce complex balance behavior to a limited set of physiologically meaningful parameters. If successful, the parameters and the underlying control model quantitatively reproduce the experimentally assessed balance behavior.

The most prominent model in the literature is the ’Independent-Channel’ model proposed by Peterka^1^. A more recent update includes a detailed description of the model and the required techniques for a single-subject characterization of the balance control mechanism^5^. For visual scene perturbations, the focus of the current study, the model contains five parameters that are optimized to reproduce individual subjects’ sway responses to perturbations. The sensory integration is a weighted sum of all sensory contributions. The visual weighting parameter $W_v$ gives a percentage of the visual contribution to the overall torque. The rest ($100\% - W_v$) is generated by the proprioceptive and the vestibular contributions, which cannot be separated without additional perturbations. The parameters $K_p$ and $K_d$ are the proportional and the derivative feedback gain, i.e. the strength of the muscle contraction relative to the deviation from the desired upright position and body sway velocity, respectively. The feedback time delay parameter $\tau$ accounts for all time delay components (neural conduction times, muscle activation, etc.) in the neural control mechanism^1^. Lastly, the parameter $K_t$ is a measure for the contribution from a low-pass filtered positive torque-feedback loop, proposed to explain low-frequency sway characteristics with a period $\gtrapprox 20 s$^16,17^. These parameters can be estimated from moving visual scene experiments in combination with model simulations and optimization techniques.

One major drawback of experiments using sensory perturbations as input is the required experimental setup. Motorized visual scenes (or surfaces) are costly and difficult to tune to the required precision^8^. Also, setups are often prototypes and therefore unique, making it difficult to reproduce data across labs. Various alternative experimental manipulations have been successfully used to induce sensory perturbations. Examples are tendon vibrations (proprioception)^3^, fast rotating discs^18^, optic flow pattern projected to screens^15,19,20^, real world moving rooms^21–24^ (visual) and galvanic vestibular stimulation (vestibular)^3,25,26^. The big advantage of surface or visual scene tilts is that they induce ecological conflicts, i.e. they mimic natural conflicts that occur in everyday life. Furthermore, tilt perturbations are relatively easy to model as kinematic variables describing the physical orientation between body and sensory references (surface, visual scene, etc.).

Virtual reality can be used to implement moving visual scenes as controlled sensory perturbations^15,19,27–29^. As VR hardware has become less expensive, easily accessible, as well as better in terms of field of view, time-delays and resolution, VR could substitute motorized setups and make perturbation based balance assessments universally available. Also, some VR systems have integrated tracking devices that can be used as a fully integrated motion capture system. In a recent study, we showed that in standing balance, body sway is similar when viewing a photo-realistic virtual-reality room as compared to viewing the real-world room^30^. We tested this on fixed and tilting support surfaces, but while keeping the visual scene space stationary.

**Figure 1:**
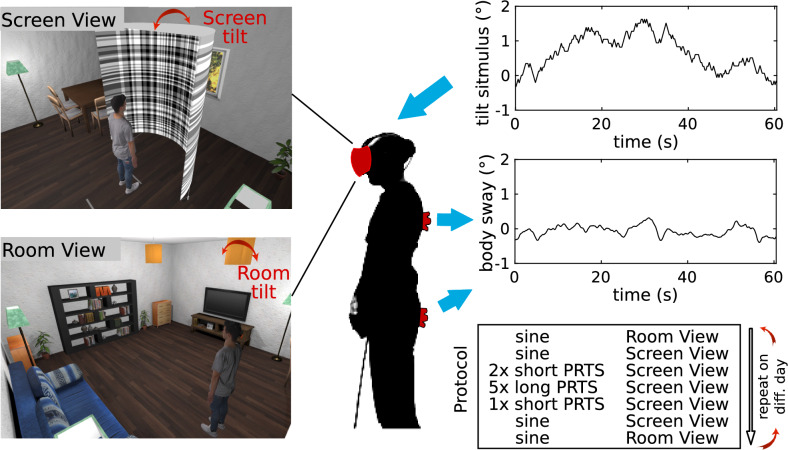
Virtual screen and room used during the experiments together with the hardware setup. The VR avatar indicates the perspective of a subject in the respective condition. Both images are screenshots of the custom made application created in Unity (Unity Technologies, San Francisco, USA). Head-mounted display used to display the virtual environment and the two VR motion tracker used for body sway recordings are shown in red. Top panel on the right shows an example of the long-PRTS stimulus sequence (60.5 s) that was repeated five consecutive times for each of the 5 amplitudes (peak-to-peak, pp0.5, pp1, pp2, pp4, pp8). Middle panel shows example whole body centre of mass sway derived from marker recordings. Bottom panel shows order of trials as applied during the experiment.

The question remains, whether moving visual scene experiments in VR are equally comparable to real-world moving scene experiments and whether they can be used for reliable balance assessments using systems identification techniques. Thus, the purpose of the current study was to validate moving scene experiments in VR. To this end, we reconstructed a moving screen from a published real-world setup^1,5^ in VR (see Fig. 1). The original experiment consisted of 60.5-s long pseudo-random ternary sequences (’long-PRTS’), which were repeated 6–8 times, each presented at 5 amplitudes. We implemented the same sequences with ten repetitions to compare our VR results to the published real world results. The first tested hypothesis was:

- H1: Sway responses to a virtual moving screen do not differ to those of a similar real-world screen.

The long-PRTS assessments required long standing periods for subjects, which is not feasible for many applied research questions^5^. We therefore also implemented a 120 s version with six repetitions of 20-s sequences (‘short-PRTS’) for a full assessment. We tested the reliability of this short version by repeating the trial twice before the long-PRTS trials and once after. We also repeated the full experiment on a second day, providing us with six repetitions, which we used for a reliability analysis. The second hypothesis was:

- H2: The short-PRTS parameter estimates do show a high Intraclass Correlation between repetitions within one session and between two sessions on two different days.

In our previous study investigating balance in a stationary virtual visual scene we found differences between sway in a photo-realistic and an abstract scene^30^. We speculated that this could be caused by subjects feeling less present in the abstract scene or by the difference in the visual input. In contrast, other studies using abstract^31^ and photo-realistic scenes^28^ found similar coherence values for sway responses to pseudo-random visual scene tilts, respectively. Therefore, there remains uncertainty how visual scene type may affect sway responses. As the screen we used in the moving screen setup was also quite abstract, we further added a comparison of sway responses to movements of the screen and a virtual room (see Fig. 1). In separate trials, room and screen were tilted following a very small sine. A sine provides much less information compared to a PRTS, however, the very short cycle duration allowed us to obtain more repetitions and therefore more averaging during short recordings. This allowed us to implement very small sine amplitudes and still obtain a reasonably reliable estimate of the response amplitude to the sine. Subjects were asked to look at the room/screen and body sway was measured during the perturbations.

- H3: Sway responses to a small sinusoidal visual scene tilt differ in dependence on the type of scene.

## Results

### VR and real-world comparison (H1)

#### General description of recorded sway responses

**Figure 2:**
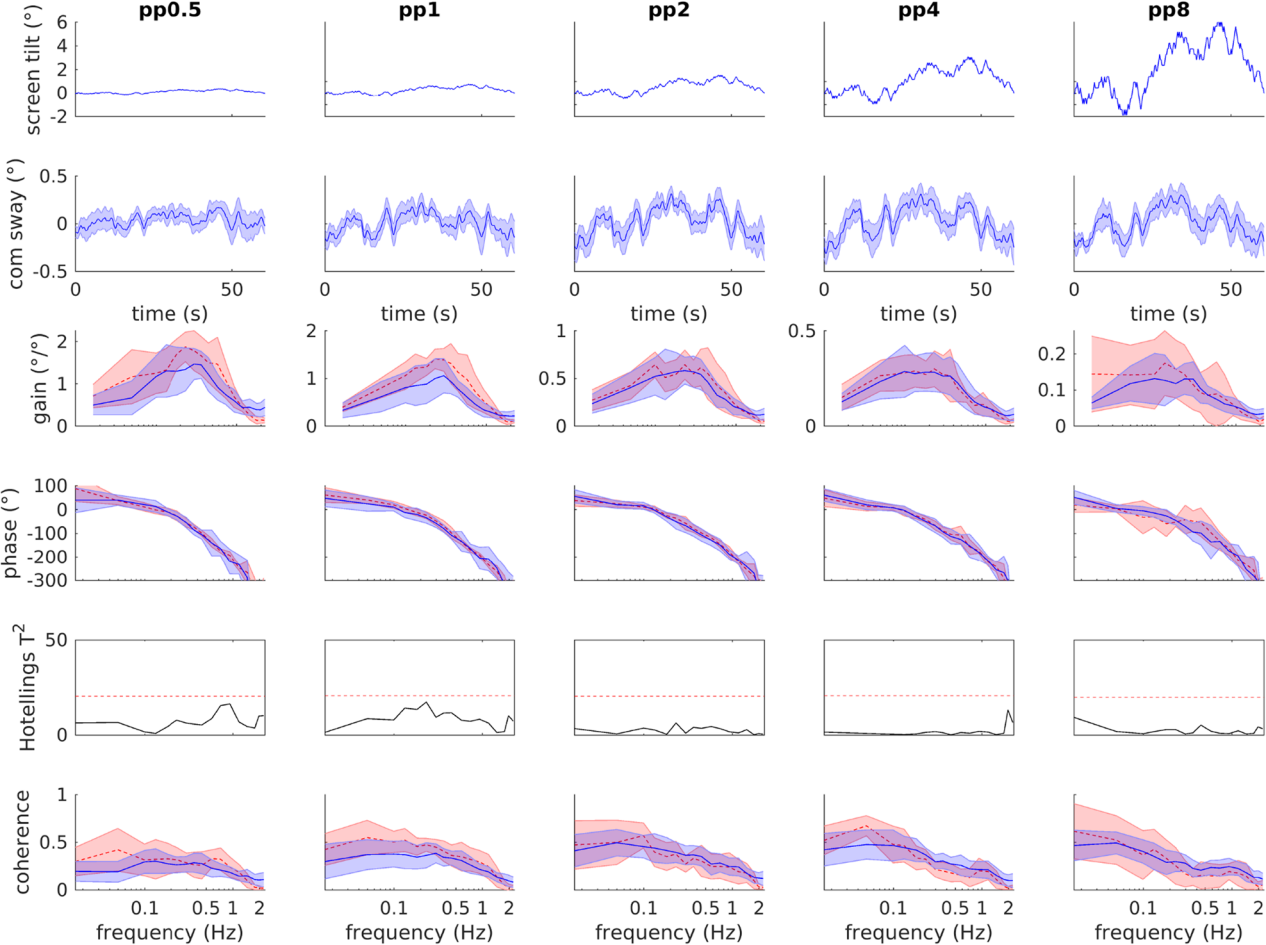
Long-PRTS sequences and sway responses of VR moving screen experiments (blue) and real-world sway responses^1^ for comparison (red), each shown as mean and $95\%$ confidence interval (shaded area). The stimulus sequence (top row) was applied at five peak-to-peak amplitudes. Each pp condition contains averages across eight repetitions of the sequence and 14 subjects. Row two shows the averaged center of mass (com) sway, rows 3 and 4 the frequency response functions (FRFs) displayed as gain and phase across frequency. Row five shows the statistical comparison of VR and real-world FRFs, where the red dashed line indicates the $\alpha =0.05$ cutoff. Bottom row shows coherence indicating the ratio between random-sway and sway evoked by the stimulus. Smaller values indicate a larger random sway component.

Figure 2 shows the results of the five long-PRTS amplitude conditions recorded in VR (blue). Screen tilt sequences are shown in the top row. The second row shows center of mass (com) sway averaged across 14 subjects and eight sequence repetitions each (four for each peak-to-peak amplitude on each day, additional first sequence was discarded to avoid transients). Com sway shows consistent response patterns to the stimulus at all amplitudes. However, compared to the large increases in stimulus amplitudes (pp$0.5^{\circ }$ to pp$8^{\circ }$), the com sway increases much less and saturates showing peak-to-peak values of $0.25^{\circ }$, $0.38^{\circ }$, $0.54^{\circ }$, $0.57^{\circ }$, and $0.50^{\circ }$, respectively. This saturation is even better visible in the frequency domain analysis. We calculated complex valued frequency response functions (FRF) and displayed them as gain and phase (Fig. 2 row 3 and 4). Gain is the amplitude ratio of stimulus and response in the frequency domain, where gain = 1 means equal stimulus and response amplitudes, gain = 2 twice as large response compared to the stimulus amplitude and gain = 0 no sway evoked by the stimulus. Recorded gain values show a strong decrease across stimulus amplitudes (note the difference in y-axes from left to right). Thus sway response amplitudes are much smaller relative to the stimulus at larger amplitude conditions. Phase values (Fig. 2 row 4) represent the temporal relation of stimulus and response, where a phase of $0^\circ$ means ’in phase’ and $\pm 180^\circ$ ‘counter phase’. Phase systematically decreased with increasing frequency, showing a phase lead of $\approx 50^\circ$ at 0.0165 Hz and a phase lag of $\approx 180^\circ$ at about 1 Hz. With increasing stimulus amplitude, phase showed slightly reduced phase lag at higher frequencies, but otherwise no systematic difference. Coherence values were overall quite low and showed a decrease towards higher frequencies. Averaged across frequency, coherence was $Coh\approx 0.21$ for pp0.5 and $Coh\approx 0.28$ at all other amplitude conditions.

#### Comparison of VR and real-world data

In the frequency domain, the real-world moving screen data of Peterka^1^ is displayed for comparison (Fig. 2 red, dashed). Overall, our VR data and the real-world data showed very similar behavior: gain values have a concave shape across frequency and decline strongly across amplitude conditions; phase values show a systematic decline across frequency and a slight decrease in phase-lag across amplitude conditions; coherence values are similarly low in both data sets. Visually, gain values did show higher sway in the real-world data, as compared to VR for peak-to-peak (pp) 0.5 and pp1. We statistically compared VR and real-world FRFs using a 1D statistical non-parametric mapping Hotellings T$\vphantom{0}^{2}$-test (Fig. 2 row 5). The statistical comparison showed no significant difference $(\alpha <0.05)$.

#### Model fit parameters

**Figure 3:**
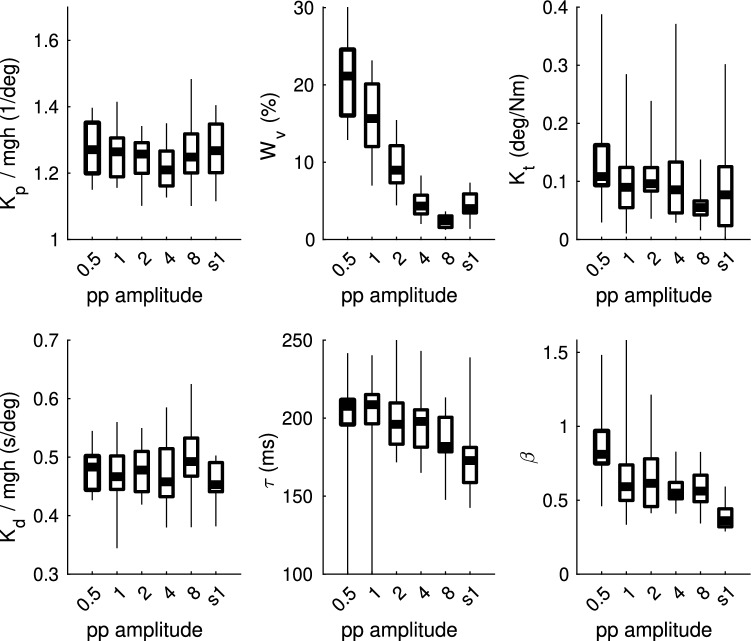
Independent-channel model parameters estimated for the five long-PRTS peak-to-peak amplitude conditions and the short-PRTS amplitude condition (s1). In addition also variance parameter $\beta$ is displayed. Parameters are shown for each individual subject (grey) and the across-subject mean, lower quartile and range (box-plots). $K_p$ and $K_d$ are normalized by mgh (subjects mass*com-height*gravitational constant).

**Figure 4:**
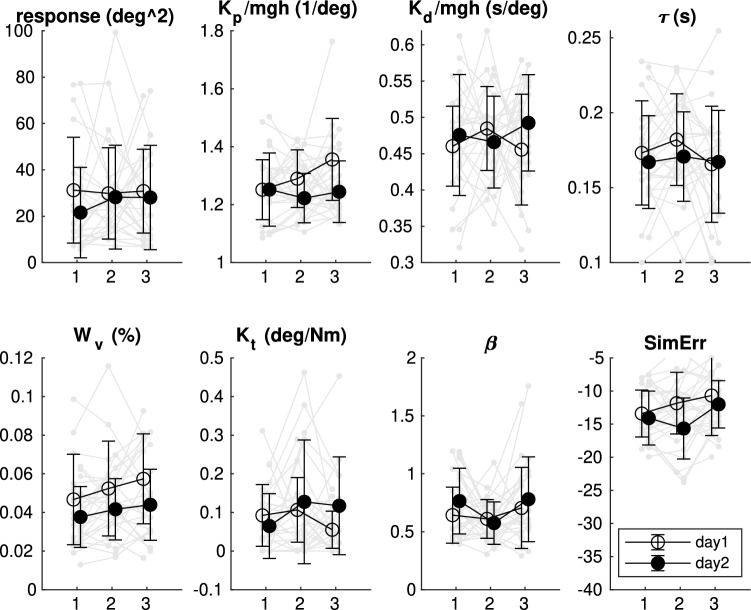
Independent-channel model parameters for each individual short-PRTS condition (pre1, pre2, post as 1–3 on x-axis). In addition also descriptive sway response power, variance parameter $\beta$ and simulation error (SimErr) are displayed. Parameters are shown for each individual subject (grey) and the across-subject mean, and standard deviation. $K_p$ and $K_d$ are normalized by mgh.

We used model based interpretations of the sway response data to extract descriptive parameters that allow intuitive interpretations (Independent-Channel Model^5^). Figure 3 shows the estimated parameters of the five long-PRTS conditions and for the average of all short-PRTS conditions (Fig. 4 ‘s1’). The visual weight ($W_v$) decreased systematically with increasing stimulus amplitude (rmANOVA; $p<0.001$, $\eta ^2=0.915$). Also, visual weight showed consistent patterns within subjects, where relatively low or high visual weights compared to other subjects were maintained across amplitude conditions. Further differences were found for $\tau$, $K_t$, and $\beta$. The parameters $\tau$ and $K_t$ decreased with increasing stimulus amplitude ($p<0.05$, $\eta ^2=0.253$ and $\eta ^2=0.269$, respectively). $\beta$ showed a large drop from pp0.5 to all other conditions ($p<0.001$, $\eta ^2=0.395$). These effects were less systematic within subject as compared to the visual weight. Parameters $K_p$ and $K_d$ showed no significant difference between pp conditions. The short PRTS parameters were compared to the pp4 condition. In pilot experiments, we found a strong similarity between the visual weight ($W_v$) values for PRTS sequences with similar velocities. To test this hypothesized stimulus velocity dependence of model parameters in favor over an amplitude or other PRTS shape characteristic, we designed the short PRTS to match the velocity of the pp4 long-PRTS condition. The statistical comparison using a paired samples Wilcoxon signed rank test showed only a significant difference for $\beta$ ($p<0.05$) and a trend towards smaller time delay values in the short-PRTS ($p=0.068$).

### Test–retest reliability

The short-PRTS condition was tested twice before and once after the five long-PRTS trials on each day to obtain test-retest reliability of the parameter estimates. Figure 4 shows the results of the five parameters together with the sway-response power, the variance parameter $\beta$ and the simulation error of the optimization procedure. Parameter estimates showed large within and between subject variability. Systematic changes were only found for the visual weight, which was slightly reduced on the second as compared to the first day (rmANOVA: $p<0.05$, $\eta ^2=0.139$). We calculated Intra-Class Correlations (ICC) to obtain test-retest reliability across all six individual measurements (Table 1, top row). ICC values were generally below 0.5, indicating poor across measurement agreement, when using 5 stimulus cycles to estimate parameters. We then pooled all short-PRTS cycles within each day and estimated parameters for the average sway response across these 15 cycles and recalculated the ICC between days (Table 1, bottom row). Here we obtained good to excellent agreement apart from the low-pass filter gain $K_t$ and the variance parameter $\beta$ which showed poor to fair agreement across days.

**Table 1:** Intraclass correlation point estimates (ICC3,1) for the independent channel model parameter estimates.

ICC3,1	$K_p$		$K_d$		$\tau$		$W_v$		$K_t$		$\beta$	
6 trials	0.11		0.14		0.24		0.46		0.21		0.15	
$95\% ci$	$-$0.03	0.39	$-$0.01	0.42	0.59	0.53	0.24	0.72	0.04	0.50	$-$0.00	0.43
d1 vs d2	0.87		0.92		0.77		0.70		0.50		0.42	
$95\% ci$	0.65	0.96	0.76	0.97	0.42	0.92	0.29	0.89	$-$0.02	0.81	$-$0.12	0.77

Top row: each of the six short-PRTS recordings of each of the 14 subjects was taken as one measurement. Bottom row: cycles of the three measurements within 1 day were pooled and ICC values calculated for day1 and day2 estimates.

### Comparison of visual scenes

**Figure 5:**
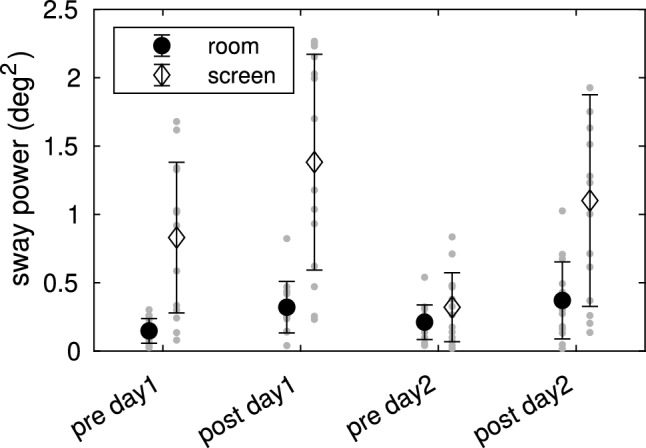
Body sway in response to visual scene tilts when viewing the room or the screen. Shown is the com sway power at the frequency of the scene tilt stimulus (0.5 Hz sine with $0.05^{\circ }$). Room view and Screen view were measured at the beginning and the end of each session (pre and post) and on 2 different days (day1, day2).

Figure 5 shows the com sway at the stimulus frequency to identical scene tilts around the ankle joints, in the screen view or the room view. Both scenes were tested at the beginning (pre) and end (post) of each session and on 2 days. The 2 $\times$ 2 $\times$ 2 rmANOVA (2 scenes, 2 days, pre-post) showed significant differences for all main effects (all $p<0.001$; scene $\eta ^2 =0.35$; pre-post $\eta ^2 =0.15$; day $\eta ^2 =0.02$), as well as for the interactions pre-post*scene and scene*day ($p<0.05$). Following our hypothesis and given the significant main effect of scene-type on our sway responses we conducted Post-hoc analysis to further investigate the observed differences between scene types (Hypothesis 3). Post-hoc comparisons of the scenes showed significantly larger sway during the screen view as compared to the room view, with a very large effect size ($p<0.001$, Cohen’s $d=1.423$).

## Discussion

We validated a virtual reality implementation of a sensory integration test using moving visual scene perturbations. We addressed three questions: (1) Are sway responses comparable to a real world setup? (2) How good is the test-retest reliability? (3) Do sway responses depend on the scene type? Our results showed that the VR implementation can be compared to the real-world implementation, as we found no differences between balance responses (Fig. 2). Also parameter estimates showed the same general pattern as described for the real-world data using a very similar model^1^. We found poor reliability across all six short-PRTS measurements. However, parameter estimates from pooled data of all three short-PRTS measurements (15 cycles) showed excellent reliability between both days with a small systematic reduction of the visual weight on the second day. Finally, we found sway power during sinusoidal tilts of the abstract screen to be three times larger as compared to tilt of the realistic room, confirming the hypothesized dependence of sway responses on scene type.

The approach we used for balance assessment depends on the model based interpretation of the data. A model always captures only specific aspects of the natural object or system. Thus, explanatory and predictive power, the usefulness to guide our understanding and research, as well as its fruitfulness in terms of applications such as diagnostic capabilities are factors by which a model should be judged. Our discussion of the proposed approach and our results will be guided by these criteria.

The IC model used for our data analysis is able to reproduce most characteristics of sway responses to real-world visual scene tilts. Our estimates using only five stimulus cycles resulted in very large variability and poor reliability of parameter estimates. Peterka et al.^5^ showed that averaging 9–14 cycles instead of the five used in our study further improves the reliability of parameter estimates for such small sway responses as during visual scene perturbations (see Fig. 4 in^5^). In agreement with this finding, we obtained much better intra-class correlations when pooling our data across all three short-PRTS measurements within each day and comparing parameter estimates of these 15 cycles between days. Therefore, we conclude that five cycles are insufficient for reliable estimates.

Interestingly, we found differences for the sine sway responses that were not present in the sway response power of short-PRTS trials: sway responses to the sine screen tilts were smaller at the beginning of a session (pre) as compared to the end (post) and smaller on the second day. Potential reasons are purely speculative, as one would expect that for any kind of learning, the changes pre-post would be in the same direction as from day1 to day2. In addition, the absence of these effects in the room view and the short-PRTS sequences appears counter intuitive. The considerably larger stimulus velocities for the short-PRTS ($\approx 0.94^\circ$/s) as compared to the very small sine stimulus amplitudes ($peak~velocity \approx 0.16^\circ$/s) might mask the effects observed in the sine conditions.

The IC model does not explain the dependence on visual scene type. We found larger sway responses to tilts of the VR screen view as compared to exactly the same tilt sequence applied to the VR room view (see Figs. 1, 5). In an earlier study, we found increased sway when viewing an abstract virtual scene as compared to a photo-realistic virtual scene^30^. Reduced spontaneous sway as found in the previous study is typically connected to a higher use of the more reliable visual input. Assuming the realism of the scene would be the modulating factor, visual input would be used less when viewing the abstract screen. Thus, the screen would evoke smaller sway responses, which is in contrast to our findings. Another explanation would be the difference in the information content of the visual scene. The VR screen was $\approx 1.2$ m in front of the ankle joint axis and therefore much closer to the subject than the walls of the room ($\approx 4.5$ m). Furthermore, the screen view contained much stronger contrasts. Equal scene tilt and/or subject sway therefore results in different optic flow patterns, which could have caused the larger evoked sway responses in the screen view. However, a more systematic analysis of the relation between optic flow properties and evoked sway is required to support this claim. Further research should also take other aspects such as the task dependence of visual effects on balance^32^ into account.

The IC model shows systematic changes of parameters across different stimulus amplitudes, showing that changes in sway responses are mostly caused by sensory reweighting. While this interpretation provides a physiologically plausible explanation, it does not provide an explanation of how reweighting is achieved by the central nervous system. Several reweighting mechanisms have been proposed in the literature for support surface tilts^14,33–35^ and movement of a touch reference^36^, as well as for visual scene tilts^37^. The implementation and comparison of such models are beyond the scope of the current study.

The implemented sensory integration test relies on visual perturbations. As such, it is able to identify the visual contribution, but cannot separate the contribution of the proprioceptive and the vestibular systems^5^. While this aspect is a considerable limitation of the proposed VR-base approach, it still provides a considerable amount of information. For example visual dependence can be estimated, as the visual weight provides a direct measure of the visual contribution to balance. This may be particularly relevant to diagnose patients with suspected increased visual dependence. Other approaches such as the Romberg ratio have limited validity, as changes in spontaneous sway patterns can have many reasons outside sensory aspects, such as attention, anxiety^38^ or stiffness^39^. Visual dependence has also been estimated using perceptual procedures (rod and frame or rod and disc test)^20^, which showed that visual dependence increases with age and is related to falls^40^. Our approach now allows researchers to assess the visual contribution during balance with minimal implementation effort.

The presented experiments and the VR implementation have several limitations. Our approach used a between-subjects design. Although our results show a good agreement with real-world experiments as well as a very good reliability, smaller differences might require a within subject comparison. Further limitations concern the technical specifications of the VR environment. VR systems are limited in terms of the field of view, the resolution and the refresh rate. For example, spontaneous sway increases, when the size of the field of view is reduced^41^. Also the delay between head motion and the update of the display likely influences the results. The technical delay likely adds to the physiological delay in some way, thereby increasing the time delay model parameter values $\tau$ by a few milliseconds. Perception in VR is affected by a vergence-accommodation conflict, as the lens of the eye needs to adjust for a fixed distance, while the convergence of the eyes varies dependent on the distance of the object in focus. This as well as potential errors in IPD adjustments and other factors are speculated to lead to a distortion in depth perception^42^. While we found no major differences to real-world balance behavior, it is likely that balance behavior and measured parameters are affected by those limitations to some extent.

Despite these limitations, virtual reality systems are widely used and have become very affordable. Furthermore, they appear to be well accepted in elderly populations^43^. As a result, with an appropriate standardized implementation, the approach validated in this study can be applied in various fields. Examples are diagnostics of neurological patients, in applied balance research to tailor interventions and test their effect on sensory integration, return to sports evaluations after concussions, assessment of sensory loss after traumatic injuries in orthopedic settings, and others.

In conclusion, our results demonstrate that the proposed virtual reality setup provides reliable estimates of the human sensory integration mechanism underlying balance. The results are comparable to real-world data. It is easy to implement and setups can be exactly reproduced across labs, making it feasible for multi-centered studies. The strength of the IC-model as the basis for data interpretations lies in its capability to separate sensory contributions and estimate the feedback dynamics of the balance control mechanism. While the analysis is complex, analysis routines can be standardized and implemented. Altogether the approach can yield as a tool to diagnose deficits in the sensory integration processes underlying balance.

## Methods

### Participants

Fourteen healthy subjects (8f/6m/0d; 23.8 ± 2.6 years; 172 ± 9 cm; 66.9 ± 9.1 kg) participated in the study. Exclusion criteria were self-reported orthopedic and neural disorders, concussions, as well as a history of epilepsy. The protocol was in agreement with the latest revision of the Declaration of Helsinki and was approved by the ethics committee of the University of Konstanz. Subjects were informed about the purpose and procedures of the study, gave written informed consent prior to participation, and were paid 10€/h in the lab.

### Experimental setup

The experimental setup is shown in Fig. 1. Subjects wore a head mounted display (HMD; Vive Pro Eye, HTC, Taoyuan, Taiwan), which has a $110^{\circ }$ field of view, a refresh rate of 90 Hz and a resolution of 1440 $\times$ 1600 pixels per eye. Vive Trackers (HTC, Taoyuan, Taiwan) were attached to hip and shoulder using velcro straps and 4 lighthouses (SteamVR Base Station 2.0, HTC, Taoyuan, Taiwan) were positioned in the corners of a 5 $\times$ 5 m room. Tracker and HMD positions were recorded at every screen update ($\approx 90 \,\hbox {Hz}$). The virtual environment, recording of tracker and HMD positions, and a user interface to run the experiments were implemented in a custom application developed with Unity (Unity Technologies, San Francisco, USA) and SteamVR (Valve, Bellevue, USA). The virtual environment consisted of a living room 6x8 m containing a half-cylindric screen (radius 1 m) with vertical and horizontal stripes (Fig. 1). The application was able to move the room or the screen in six dimensions (3 rotations, 3 translations), following predefined sequences.

During experiments, the visual scene was tilting around the ankle joints in anterior-posterior direction (axis of rotation was 8.8 cm above the floor). The visual scene was either the living room (Room View), or the Screen View, where subjects saw nothing but the screen when looking straight at the screen (Fig. 1). We used three stimulus types, a sine ($0.05^{\circ }$; 0.5 Hz) and two types of pseudo-random-ternary sequences (PRTS). Stimulus sequences containing a sine started and ended with a raised cosine to avoid big accelerations. The PRTS alternates a fixed positive, negative, or zero velocity^1,44^. The short PRTS had 80 states with a state duration of 0.25 s and was repeated six times at a peak-to-peak amplitude of $2.1^{\circ }$, resulting in 120 s trials which were superimposed with a linearly independent sine. The superimposed sine was not used in later analyses. The long PRTS sequence had 242 states with 0.25 s state duration and was identical to the stimulus used by Peterka^1^. The 60.5-s long sequence was repeated five consecutive times resulting in $\approx 5$ min long trials. Five 5 min long trials were tested, each at a different peak-to-peak (pp) amplitude: 0.5; 1; 2; 4; and $8^{\circ }$. In summary, three different stimulus sequences were applied (1) sine only (either room or screen tilt); (2) short-PRTS (screen tilt); (3) long-PRTS at five amplitudes (screen tilt).

### Procedures

After signing the informed consent, subjects filled out a basic health questionnaire and mass and height were measured. The distance between the lenses of the HMD, as well as the render camera separation were adjusted to the subjects interpupillary distance (IPD). IPD was measured using the procedure suggested by the manufacturer of the VR device. Subjects were asked to close one eye and align a scale to the pupil of the open eye using a mirror. Then, closing the other eye, they were asked to read of the distance of the other pupil from the scale. Velcro straps with the trackers were attached and subjects were asked to put on the HMD, which already showed the living room. Subjects were given a short period of time (2–3 min) to familiarize with the virtual environment, but were asked to not walk more then 1 m from the starting position. For the room view, subjects were asked to stand on a line on the floor. Foot position was corrected if necessary to align the ankle joints with the axis of rotation. For the screen view, subjects were asked to turn around and face the screen, again positioning the feet. For the sine trials (first two and last two on each day), subjects were not told about the scene movement and asked to “stand upright and comfortable” and to “look straight ahead” for a 2-min long quiet stance recording. For the short-PRTS and the long-PRTS trials subjects were told that the screen would move and were asked to “stand upright and comfortable” and to “look straight ahead”. Subjects listened to non-rhythmic audio books during all recordings to distract from the balancing task and avoid auditory orientation. Subjects were given short breaks to move in between trials and were allowed to take off the HMD and sit down for longer breaks upon request. The whole procedure took 60-90 min and subjects were asked to come again for a second recording on another day.

### Preprocessing of data

Data was saved as CSV files and further analyzed in Matlab (The Mathworks, Natick, USA). As the recording was coupled to the refresh rate of the displays, the actual sampling rate was 11.11 ± 0.29 ms. Data was resampled to exactly 90 Hz using the Matlab function ‘resample’, before further processing. Whole body center of mass tilt around the ankle joints (com) in anterior–posterior direction was used as the primary variable for all analyses. We used the shoulder and the hip marker to approximate the com for all recordings, assuming two segment biomechanics with ankle and hip joints. Leg and trunk segment orientations were calculated from the anterior–posterior shoulder and hip marker positions and the com angle was calculated thereof using subjects anthropometrics and mass distribution tables^45^. As the shoulder marker dropped out in four recordings of one subject (battery issues), we estimated the trunk movement from head (HMD) movements for these trials. As it would simplify the setup if using the HMD instead of an additional shoulder marker, we estimated the validity of such an approximation. Trunk sway estimated from relative hip-to-shoulder marker movement was considerably larger as compared to an estimate from hip-to-head movement. We believe that the small error is justified in our case due to the small number of approximations. However, our results indicate that the shoulder marker typically used for com estimation cannot generally be substituted by HMD movement.

### Analysis of long-PRTS sequences

The first of the five PRTS sequence repetitions was discarded to avoid transients and the 2 $\times$ 4 cycles for each subject were transformed to the frequency domain using a Fast Fourier Transform (Matlab function ‘fft’). Frequency response functions (FRF) were calculated by dividing the averaged com spectrum by the averaged stimulus spectrum, discarding even frequency points, where the stimulus has no energy, and averaging across frequency to reduce the number of frequency points at higher frequencies^1^. Coherence was calculated as the product of the averaged power spectra of com and stimulus, divided by the averaged cross-power spectrum of com and stimulus (for details see^1,5,46^). FRFs are complex valued functions of frequency and were displayed as gain and phase, where gain is the ratio of com sway and stimulus amplitudes, while phase gives their temporal relation. Coherence is a measure of the relation between the random and stimulus evoked com sway components. A coherence of one would indicate no random and only stimulus evoked sway, while a coherence of zero would indicate only random and no stimulus evoked sway.

The long PRTS data from our VR experiments was compared to the data set recorded by Peterka^1^ using a motorized real-world screen. Single subject FRFs from both studies were used for the between-subject statistical comparison. FRFs are complex valued, which was accounted for using two-sided $Hotellings-T^2$ tests treating real and imaginary components as dependent variables. As FRFs are one-dimensional across frequency, i.e. they contain many frequency points, the test statistic needs to be corrected for multiple hypothesis-testing. As the FRF data-points are not independent, there are fewer degrees of freedom as the number of frequency points. We used the statistical non-parametric mapping package (spm1d.org version M.0.4.8), which applies random-field theory to calculate alpha-level adjustments for 1D data^47–49^. In summary, we calculated two-sided non-parametric $1D- Hotellings-T^2$ tests with an alpha level of $\alpha =.05$ for the frequency-dependent FRFs to compare the Peterka^1^ (real-world) and our VR data at each stimulus amplitude.

### Parametric analysis of long-PRTS and short-PRTS sequences

Frequency response functions of the short-PRTS sequences were calculated analogous to those of the long-PRTS sequences and again averaged across frequency as proposed by Peterka^5^. The FRFs were interpreted using a parametric model, where the sensory integration mechanism is modeled as a feedback mechanism, formulated as a differential equation. Optimization techniques are then used to identify the parameters that best reproduce the experimental FRFs. Model formulation and parameter estimation was performed in large part following the methods described by Peterka^5^. The model dynamics are given by:

1 $$\begin{aligned} H_m(\theta ,k) = \frac{W_v \cdot NC \cdot TD \cdot B}{1 - TF \cdot NC \cdot TD + NC \cdot TD \cdot B } \end{aligned}$$

with the linearized (small angle approximation $\sin (\gamma )\approx \gamma$) body dynamics $B=\frac{1}{Js^2-mgh}$, the neural controller $NC=K_p + K_d s$, the time delay $TD = \exp ^{-\tau s}$, the low-pass torque feedback $TF=\frac{K_t}{sF_{lp}+1}$, and the Laplace variable $s(k)=j\omega (k)$. Body inertia J and mass*gravitational constant*com height (mgh) were calculated from subjects weight and height and anthropometric tables^45^. We used a fixed $F_{lp}=20$, which differed to the methods described by Peterka, who estimated $F_{lp}$^1^ for the long-PRTS sequences or proposed to use $TF=\frac{K_t}{s}$ for the short-PRTS sequences. The short-PRTS sequences are too short for reliable estimates of $F_{lp}$, while a pure integrator is not performing very good for the long-PRTS sequences. Thus we chose the fixed $F_{lp}$ based on an estimate from the literature^16^. Model parameters subject to optimization are thus $\theta =(W_v,K_p,K_d,K_t,\tau )$.

We propose a Maximum-Likelihood estimator assuming a normalized Laplace-distribution to formulate the parameter estimation problem. The Laplace distribution is given by:

2 $$\begin{aligned} p(x|\mu ,b) = \frac{1}{2b} \exp^{-\frac{|x-\mu |}{b}} \end{aligned}$$

The probability to observe the experimental FRF $H_e(k)$ for any model FRF $H_m(\theta ,k)$ is then given by:

3 $$\begin{aligned} p(H_e(k)|H_m(\theta ,k)) = \frac{1}{2\beta |H_m(\theta ,k)|} \exp ^{-\frac{|H_e(k)-H_m(\theta ,k)|}{\beta |H_m(\theta ,k)|}} \end{aligned}$$

The negative log-likelihood is then given by

4 $$\begin{aligned} -\log (p(H_e(k)|H_m(\theta ,k))) = \log (2\beta |H_m(\theta ,k)|) + \frac{|H_e(k)-H_m(\theta ,k)|}{\beta |H_m(\theta ,k)|} \end{aligned}$$

The parameter optimization problem can then be formulated as

5 $$\begin{aligned} {\mathop {\textrm{minimize}}\limits _{\theta , \beta }} \sum _{k=0}^{N-1} \log (2\beta |H_m(\theta ,k)|) + \sum _{k=0}^{N-1} \frac{|H_e(k) - H_m(\theta ,k)|}{\beta |H_m(\theta ,k)|} \end{aligned}$$

Statistical comparisons of parameters were implemented in JASP^50^. For repeated measures ANOVAs, we used the assumption checks (Mauchly’s test for sphericity) and appropriate corrections (Greenhouse–Geisser) where appropriate. Also we tested for normality (Shapiro–Wilk) in pairwise comparisons and used the Wilcoxon signed-rank test for all comparisons as normality assumption was violated in some parameters. Reliability analysis was performed using the intraclass correlation (ICC3,1) implemented in JASP^50^.

### Analysis of sine sequences

Sway response amplitudes at the sine stimulus frequency were calculated for each subject and condition. Com sway data was transformed using a fast-fourier transform (‘fft’) and the half-sided power-spectrum was scaled (1/Fs/N; N $=$ samples in the time domain; Fs = sampling rate). Finally, the value at the stimulus frequency (0.5 Hz) was taken for statistical comparisons across conditions. Sway responses of the sine conditions were statistically compared using a 3-level rmANOVA with the levels ‘scene’ (Room, Screen), ‘pre-post’ (pre, post), and ‘day’ (day1, day2).

## Data Availability

The data is available from L.A. upon reasonable request.
